# Automating the Addiction Behaviors Checklist for Problematic Opioid Use Identification

**DOI:** 10.1001/jamapsychiatry.2025.0424

**Published:** 2025-04-09

**Authors:** Angus H. Chatham, Eli D. Bradley, Vanessa Troiani, Donielle L. Beiler, Parker Christy, Lori Schirle, Sandra Sanchez-Roige, David C. Samuels, Alvin D. Jeffery

**Affiliations:** 1 Vanderbilt University School of Nursing, Nashville, Tennessee; 2 Research Institute, Geisinger Commonwealth School of Medicine, Scranton, Pennsylvania; 3 Department of Anesthesiology, School of Medicine, Vanderbilt University, Nashville, Tennessee; 4 Department of Psychiatry, University of California San Diego, La Jolla; 5 Division of Genetic Medicine, Department of Medicine, Vanderbilt University Medical Center, Nashville, Tennessee; 6 Institute for Genomic Medicine, University of California San Diego, La Jolla; 7 Department of Molecular Physiology and Biophysics, Vanderbilt Genetics Institute, Vanderbilt University, Nashville, Tennessee; 8 Department of Biomedical Informatics, Vanderbilt University Medical Center, Nashville, Tennessee

## Abstract

**Importance:**

Individuals whose chronic pain is managed with opioids are at high risk of developing an opioid use disorder. Electronic health records (EHR) allow large-scale studies to identify a continuum of problematic opioid use, including opioid use disorder. Traditionally, this is done through diagnostic codes, which are often unreliable and underused.

**Objective:**

To determine whether regular expressions, an interpretable natural language processing technique, could automate a validated clinical tool (Addiction Behaviors Checklist) to identify problematic opioid use.

**Design, Setting, and Participants:**

This cross-sectional study reports on a retrospective cohort with data analyzed from 2021 through 2023. The approach was evaluated against a blinded, manually reviewed holdout test set and validated against an independent test set at a separate institution. The study used data from Vanderbilt University Medical Center’s Synthetic Derivative, a deidentified version of the EHR for research purposes. This cohort comprised 8063 individuals with chronic pain, defined by diagnostic codes on at least 2 days. The study team collected free-text notes, demographics, and diagnostic codes and performed an external validation with 100 individuals with chronic pain from Geisinger, recruited from an interventional pain clinic cohort.

**Main Outcomes and Measures:**

The primary outcome was the evaluation of the automated method in identifying patients demonstrating problematic opioid use and its comparison with manual medical record review and opioid use disorder diagnostic codes. Methods with F1 scores were evaluated (a single value that combines sensitivity and positive predictive value at a single threshold) and areas under the curve (a single value that combines sensitivity and specificity across multiple thresholds).

**Results:**

Among the 8063 patients in the primary site (5081 female [63%] and 2982 male [37%]; mean [SD] age, 56 [16] years) and 100 patients in the validation site (57 female [57%] and 43 male [43%]; mean [SD] age, 54 [13] years), the automated approach outperformed diagnostic codes based on F1 scores (0.73; 95% CI, 0.62-0.83 vs 0.08; 95% CI, 0.00-0.19 at the primary site and 0.70; 95% CI, 0.50-0.85 vs 0.29; 95% CI, 0.07-0.50 at the validation site) and areas under the curve (0.82; 95% CI, 0.73-0.89 vs 0.52; 95% CI, 0.50-0.55 at the primary site and 0.86; 95% CI, 0.76-0.94 vs 0.59;95% CI, 0.50-0.67 at validation site).

**Conclusions:**

This automated data extraction technique may facilitate earlier identification of people at risk for and who are experiencing problematic opioid use, and create new opportunities for studying long-term sequelae of opioid pain management.

## Key Points

**Question:**

Could the Addiction Behaviors Checklist be automated to efficiently identify problematic opioid use in electronic health record notes?

**Findings:**

In this cohort of patients with chronic pain, the automated approach achieved high sensitivity and positive predictive value compared with a manual review and performed significantly better than diagnostic codes.

**Meaning:**

In this study, an automated approach for the Addiction Behaviors Checklist based on clinical notes performed better than diagnostic codes and can be generalizable to any electronic health record system.

## Teaser

This study attempts to determine whether regular expressions, an interpretable natural language processing technique, could automate a validated clinical tool (Addiction Behaviors Checklist) to identify problematic opioid use.

## Introduction

Chronic pain affects more than 40 million individuals in the US, of which approximately 10 million experience high-impact chronic pain affecting daily activities^.^^1^ While current recommendations suggest a multimodal approach to chronic pain management, prescription opioids have historically been a primary treatment and continue to be used.^2^ Given the highly addictive nature of opioids, the risk of developing an opioid use disorder (OUD) is estimated to be high at approximately 18%.^3^ OUD is associated with a financial burden of more than $1 trillion when accounting for health care, lost work productivity, and criminal legal costs.^4^ To address this problem from a health care perspective, we must first be able to identify which patients experience OUD and/or are at risk for developing OUD.

The magnitude of this problem necessitates large-scale data sources for identifying individuals across the continuum of problematic opioid use. Currently, the largest source of health data are electronic health records (EHR) used for routine clinical care. The standard method for detecting a clinical problem in the EHR is through diagnostic indicators, such as problem lists or *International Classification of Diseases* (*ICD*) codes used for billing purposes.^5,6^ However, *ICD* codes are not a reliable source of OUD diagnosis because the codes are often underused, which has been attributed to OUD–related stigma and health care professional concerns about barriers to future pain management.^3,7,8,9,10^ In fact, OUD *ICD* codes have been reported to have a sensitivity of only 0.17 and a positive predictive value of only 0.58 to 0.62.^11,12,13^

Expanding the search to additional areas that contain clinical notes has been explored. Palmer et al^14^ analyzed clinical notes using natural language processing (NLP) based on matching terms in a customized dictionary of 1248 problematic opioid use key words developed by subject matter experts. They discovered NLP techniques could identify many individuals with problematic opioid use who did not have relevant *ICD* codes; however, they also found many patients with relevant *ICD* codes who were not flagged by NLP methods.^14^ Similar results were reported by Carrell et al,^15^ who developed a customized dictionary (of 1288 unique terms) based on recommendations from subject matter experts and iterative reviews of example text. The results from these articles would suggest limitations to a customized dictionary approach to NLP (or an inadequacy of EHR documentation).

The Addiction Behaviors Checklist^16^ (ABC) is a valid and reliable instrument that can be used for identifying OUD risk among patients with chronic pain.^16^ The ABC collects risk information provided by clinicians, making it a particularly suitable tool to adapt to EHR data. We used the ABC instrument instead of other OUD risk-assessment tools (eg, the Revised Opioid Risk Tool^17^) because the ABC collects risk information from language patterns used by clinicians, who are the primary authors of EHR notes. Use of such an assessment tool could guide the NLP methods for automatically searching the clinicians’ notes within the EHR. NLP can be leveraged for automating information extraction from text documents and includes techniques ranging from pattern matching to concept extraction (using specific software to identify structured medical concepts from unstructured text) to advanced numerical vector embeddings (a complex method of representing text elements in a high-dimensional space, such as that used in generative artificial intelligence).^18^

### Objective

If an NLP method that is highly interpretable by clinicians, such as one that facilitates review of matching text, could perform comparably with manual medical record reviews, there is potential to expedite and scale the identification of addictive behaviors within EHRs. Such a method could eventually be embedded within EHRs for early identification of patients at risk for OUD and facilitate follow-up care. As an initial step toward this long-term goal, in this study we sought to develop and evaluate an interpretable NLP technique to automate the ABC instrument for purposes of expediting research or clinical chart reviews.

## Methods

This study follows the Strengthening the Reporting of Observational Studies in Epidemiology (STROBE) reporting guidelines.^19^ We acquired ethics approval under institutional review boards from Vanderbilt University Medical Center (VUMC) and Geisinger. Given the deidentified nature of VUMC Synthetic Derivative, we received a waiver of informed consent. Patients at Geisinger completed written informed consent. All code is publicly available online.^20^

### Cohort Definition and Data Collection (Primary Site)

We conducted a retrospective, observational cohort study of individuals with chronic pain. We selected this phenotype because individuals with chronic pain are known to have a higher incidence of opioid use and OUD than the general population.^21,22^ We derived the study data from VUMC’s Synthetic Derivative, a deidentified version of the EHR for research purposes.^23^ Chronic pain was defined by *ICD* codes (eg, 338.2, 338.21, G89.2, G89.21; eAppendix 1 in Supplement 1 has full list) occurring on at least 2 different days to decrease false positives when including patients with only a spurious code.^24^ We collected all EHR free-text notes (eg, progress notes, patient communication, history, and physical). We collected demographic information (age, gender, ethnicity, race) from the EHR to characterize our population; ethnicity and race were self-reported for any visit starting in 2017 while there could be a mix of self-reported and health care professional–reported values prior to 2017. We limited the cohort to individuals aged 13 years or older at the time of their first chronic pain diagnosis.

### Cohort Definition and Data Collection (Validation Site)

For the external validation study at Geisinger, we selected patients who were treated at the time of consent in Geisinger’s interventional pain setting, a multisite specialty clinic that focuses on treatment of patients with complex chronic pain. These patients were enrolled in an independent prospective study related to opioid use. Different from the primary site (VUMC), we had already limited inclusion to individuals with at least 2 lifetime opioid prescriptions. Additionally, because the study at the validation site included an emphasis on genetics, we only had access to individuals of European ancestry.^25^ We selected a randomized group of 100 patients from the larger cohort and confirmed they had at least 1 *ICD* code for chronic pain based on the VUMC criteria. We collected all available free-text notes for a patient, as well as demographic information from the EHR. The parent study required a patient to be at least 18 years of age to enroll, limiting the Geisinger cohort to adults.

### Instrument Development (Primary Site)

We used the ABC^16^ to guide regular expression development. Regular expressions are a well-established method of representing a specified target text by a sequence of text operations that is designed to capture expected variation in how that target text could be presented in a free text.^18^ For each item in the ABC checklist, 3 members of the VUMC research team (a health sciences undergraduate student [A.H.C.], a biomedical data scientist [E.D.B.], and a PhD nurse practitioner and informatician [A.D.J.]) generated 1 or more regular expressions to represent the conceptual intention of the item. After drafting a regular expression, 2 separate members of the research team (a pain and opioid researcher [L.S.] and a substance use disorder geneticist [S.S.R.]) manually reviewed performance of the candidate expression by examining 50 to 100 positive matches in the training dataset.

Following a review of matches, we examined whether additional filtering for matches near 133 opioid-related terms (eAppendix 2 in Supplement 1) and/or at least 7 negation detection terms (eAppendix 3 in Supplement 1) improved performance. For example, with ABC item 2 (“Patient has hoarded meds.”), we used regular expressions to search for “hoard” and then filtered to include only those variations of “hoard,” “stash,” “left over,” “storing,” and “stockpil” (sic) that were followed by variations of “pain med,” “opioid,” “opiod,” (sic) “narc,” “analges” (sic), or an opioid drug name. Then, of those sentences, any that included a negating term preceding “hoard” (or 1 of the related verbs) were excluded. We added a final step for some expressions where we included common false-positive matches. For example, opioids were frequently mentioned in the discharge instructions of a patient’s medical record. We removed opioid matches if they were preceded by the phrase “discharge instructions.”

After a candidate regular expression’s matches were reviewed in the training dataset, modifications were made to these expressions based on suggestions from 2 of us (L.S. and S.S.R.). New examples of matches were generated by 3 of us (A.H.C., E.D.B., and A.D. J.) in another group of 50 to 100 randomly selected matches. The iterative process resulted in 27 regular expressions (eAppendix 4 in Supplement 1) representing the ABC items. We implemented the regular expressions in Python (Python Software Foundation) version 3.10. We applied each regular expression to every clinical note. If 1 or more matches for a given ABC item were discovered in any of the patient’s notes within the time window that defined the cohort, that patient received 1 point toward an overall total score.

### Instrument Deployment (Validation Site)

A software developer at the primary site and a data scientist at the validation site focused on updating the instrument’s data preprocessing and input methods without altering any of the regular expressions. The complete software code is publicly accessible in a GitHub repository where anyone can freely access the instrument.^20^

### Medical Record Review Process (Primary Site)

We evaluated our methods against a manual review in a holdout test set of 100 patients that had been adjudicated in our previous study^22^ at VUMC where we classified individuals as having no (n = 49), some (n = 33), or high (n = 17) evidence of OUD and substance use disorders (SUD). These 100 patients (of which 1 patient did not have associated clinical notes) were randomly selected from the chronic pain cohort and were only used for evaluating our phenotyping methods. We reviewed patients’ records guided by a key word template based on the *DSM-5* criteria for OUD,^26^ the ABC instrument,^16^ and others’ studies focused on detecting problematic opioid use within EHR data.^14,15,27^ Two members of the research team with extensive addiction-focused medical record review experience (a nurse anesthetist and pain and opioid researcher [L.S.] and an addiction research specialist [S.S.R.]) independently reviewed patients’ records based on the key word guide. Reviewers achieved 96% concordance after refining the key word guide and discrepancies were resolved through a consensus-building process. The supplemental material associated with our prior work further describes the medical record review process and concordance metrics.^22^

### Medical Record Review Process (Validation Site)

At Geisinger, our manual review was completed by trained study staff using a published medical record review procedure and rubric that follows the *DSM-5* criteria for OUD^27^ and assigns a severity score, ranging from 0 to 11^25^ and further adapted for use beyond chronic pain populations.^28^ Individual reviewers were research assistants trained in medical terminology and OUD symptoms, and periodic reliability checks occurred to ensure consistency across reviewers. A reliability check of 50 medical records showed an overall reliability of 91.9%. Patients included in this cohort had their medical records reviewed according to data that existed up to and including date of consent (ranging from April 4, 2018, through May 20, 2019). For algorithm generalization, notes evaluated were also limited to the date of the individual’s consent into the study, which would match the notes and information available for the manual medical record review.

### Statistical Analysis

We calculated the sensitivity (ie, recall—proportion of cases with an NLP match), specificity (proportion of controls without an NLP match), positive predictive value (ie, precision-proportion of NLP matches that were cases), negative predictive value (proportion of NLP nonmatches that were controls), and F1 score (a single measure of predictive performance combining sensitivity and positive predictive value at a single threshold) of our regular expression scoring system against the manually adjudicated labels (n = 100 at each site).

We used recall-precision curves and area under the receiver operating characteristic curves (AUCs) to evaluate performance of both the total ABC score and the OUD *ICD* codes against the manual reviews. While AUCs are frequently used in the biomedical and clinical literature to represent performance of a diagnostic test/model, reporting an AUC depends on calculating specificity, which can be misleading in information retrieval studies, such as those using NLP methods. By focusing on sensitivity (recall) and positive predictive value (precision), the measures emphasize identification of positive cases, which can be particularly helpful when datasets do not have a case-control balance.

At the primary site (VUMC), we calculated 2 additional metrics to provide further evaluation of our automated approach. First, we examined the pairwise φ coefficients (a measure of agreement for binary variables) between each item of the ABC instrument against all other items of the ABC instrument in the entire dataset. We also compared the presence of OUD and SUD *ICD* codes (eAppendix 1 in Supplement 1) present in individuals’ records.

## Results

### Cohort Description (Primary Site)

The full primary cohort comprised 8063 patients with chronic pain, of which 161 patients (2.0%) had no associated notes based on search criteria. The dataset comprised 3 482 063 accompanying notes (Table 1). A total of 1329 patients (16.5%) had an SUD *ICD* code on least 2 days while 714 patients (8.9%) had OUD *ICD* code on at least 2 days.

**Table 1.** Descriptive Statistics of Demographic Characteristics for the Patient Records Included in the Primary and Validation Sites

Variable	VUMC (primary) (n = 8063)		Geisinger (validation) (n = 100)	
	Mean (SD)	Median (IQR)	Mean (SD)	Median (IQR)
Age at earliest chronic pain diagnosis, y	56.2 (16.3)	58.0 (46.3-68.5)	53.9 (12.5)	56.0 (44.5-64.0)
Note counts	434.7 (992.4)	178 (59-473)	1568.5 (1227.8)	1246 (818.5-1958)
Gender, No. (%)				
Female	5081 (63.0)	NA	57 (57.0)	NA
Male	2982 (37.0)	NA	43 (43.0)	NA
Ethnicity,^a^ No. (%)				
Hispanic/Latino	135 (1.7)	NA	2 (2.0)	NA
Non-Hispanic/Latino	7898 (98.0)	NA	98 (98.0)	NA
Unknown	30 (0.4)	NA	NA	NA
Race,^a^ No (%)				
Asian	76 (1.0)	NA	NA	NA
Black	1336 (16.6)	NA	NA	NA
White	6499 (80.6)	NA	100 (100.0)	NA
Other^b^ or more than 1 race	122 (1.5)	NA	NA	NA
Unknown	30 (0.4)	NA	NA	NA

Abbreviations: ABC, Addiction Behaviors Checklist; *ICD, International Classification of Diseases (9th and 10th editions)*; NA, not applicable; OUD, opioid use disorder; SUD, substance use disorder; VUMC, Vanderbilt University Medical Center.

^a^ At the Vanderbilt University Medical Center site, ethnicity and race were self-reported for any visit starting in 2017 while there could be a mix of self-reported and health care professional–reported values prior to 2017.

^b^ Includes American Indian or Alaska Native, Native Hawaiian or Other Pacific Islander, none, or decline to answer.

The manually reviewed holdout test set comprised 100 patients, of which 99 patients had associated clinical notes. Fifty of these 99 patients had evidence of OUD based on manual review (Table 2). The prevalence of OUD *ICD* codes (on at least 2 separate days) among individuals with some or high evidence of OUD was small (3.0% and 5.9%, respectively; Table 2). The prevalence of *ICD* codes for the more generic condition of SUD was higher than that of OUD (15.2% and 47.1%, respectively; Table 2).

**Table 2.** Prevalence of Opioid Use Disorder (OUD) and Substance Use Disorder (SUD) *International Classification of Diseases (9th and 10th Editions*) (*ICD*) Codes Among Patients in the Primary Site (Vanderbilt University Medical Center) Test Set, Stratified by Degree of OUD Evidence on Manual Review

Degree of evidence^a^ on manual review	No. (%)	
	At least 2 d of *ICD* code present for OUD (n = 2)	At least 2 d of *ICD* code present for SUD (n = 14)
No evidence (n = 49)	0	1 (2.0)
Some evidence (n = 33)	1 (3.0)	5 (15.2)
High evidence (n = 17)	1 (5.9)	8 (47.1)

^a^ The degree of evidence ratings were based on the manual medical record review process used and described in our prior publication.^22^

### Cohort Description (Validation Site)

The validation cohort comprised 100 patients with chronic pain, all of whom had notes present. The dataset comprised 156 850 accompanying notes (Table 1). Twelve patients (12%) had an SUD *ICD* code on at least 2 days while 6 patients (6%) had an OUD *ICD* code on at least 2 days.

### ABC Performance (Primary Site)

Of the 20 ABC items, 15 items were associated with positive matches in the test set (eAppendix 5 in Supplement 1 contains item-level performance). The item-level pairwise φ correlation coefficients from the ABC instrument yielded values between –0.01 through 0.32, indicating low item-level correlation in the entire dataset. Using a total ABC score threshold of 2 or more points and combining the manual review categories of “some evidence” and “high evidence” to create binary indicators, the best balance between sensitivity and positive predictive value was achieved, which resulted in an F1 score of 0.73 (95% CI, 0.62-0.83). The total ABC score achieved an AUC of 0.82 (95% CI, 0.73-0.89) compared with manual review (Figure). The OUD *ICD* codes achieved an F1 score of 0.08 (95% CI, 0.00-0.19) and an AUC of 0.52 (95% CI, 0.50-0.55) (Figure) compared with the manual review. Therefore, the F1 score and AUC of the total ABC score outperformed the *ICD* codes.

**Figure.**
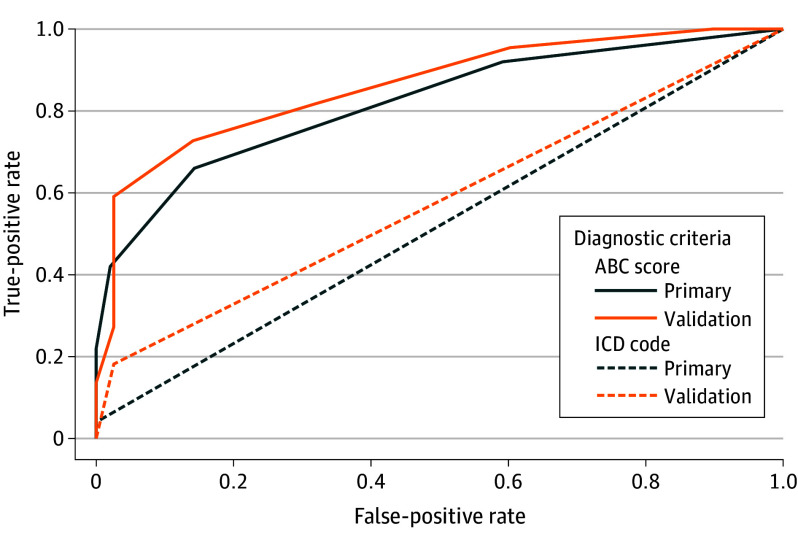
Area Under the Receiver Operating Characteristic Curve of the Automated Addiction Behaviors Checklist (ABC) Instrument Compared With Manual Review and *International Classification of Diseases* (*ICD*) Codes

As the total ABC score increased, the proportion of individuals with an OUD *ICD* code on at least 2 separate days increased (Table 3). The eFigure in Supplement 1 illustrates the sensitivity (recall) vs positive predictive value (precision) of both the ABC score and OUD *ICD* codes as compared with the manual review.

**Table 3.** Prevalence of Opioid Use Disorder (OUD) *International Classification of Diseases (9th and 10th Editions*) (*ICD*) Codes in Patient Records at Both Sites, Stratified by Total Addiction Behaviors Checklist (ABC) Score

ABC score	VUMC (primary)		Geisinger (validation)	
	Sample size	At least 2 d of *ICD* code present for OUD, No. (%)	Sample size	At least 2 d of *ICD* code present for OUD, No. (%)
0	1699	14 (0.8)	0	0
1	3145	125 (4.0)	8	0
2	1435	182 (12.7)	24	0
3	790	99 (12.5)	25	1 (4.0)
4	396	90 (22.7)	16	2 (12.5)
5	200	74 (37.0)	12	2 (16.7)
6	116	54 (46.6)	7	0
7	60	33 (55.0)	5	1 (20.0)
8	33	17 (51.5)	2	0
9	9	8 (88.9)	1	0
10	10	9 (90.0)	0	0
11	5	4 (80.0)	0	0
12	2	2 (100.0)	0	0
13	2	2 (100.0)	0	0

Abbreviations: SUD, substance use disorder; VUMC, Vanderbilt University Medical Center.

### ABC Performance (Validation Site)

Of the 20 ABC items, 13 items were associated with positive matches in the Geisinger validation site (eAppendix 5 in Supplement 1 has item-level performance). Using a total ABC score threshold of 6 points to create a binary OUD indicator, the best balance between sensitivity and positive predictive value was achieved, which resulted in an F1 score of 0.70 (95% CI, 0.50-0.85). The total ABC score achieved an AUC of 0.86 (95% CI, 0.76-0.94) compared with manual review (Figure). The OUD *ICD* codes achieved an F1 score of 0.29 (95% CI, 0.07-0.50) and an AUC of 0.59 (95% CI, 0.50-0.67) (Figure) compared with the manual review. Therefore, the F1 score and AUC of the total ABC score outperformed the *ICD* codes in the validation site, too.

## Discussion

The automatic characterization of problematic opioid use from existing clinical notes could be transformative for preventive care of chronic pain and surgical patients and identification of patients with probable opioid addiction, which would address a care gap in opioid screening practices.^29,30^ The most commonly used indicators of problematic opioid use, including OUD, within EHRs are *ICD* codes, which are not only underused^11,29^ but also often applied to individuals without a confirmed OUD diagnosis.^12,13^ We have developed an automated approach that significantly outperforms *ICD* codes with respect to both sensitivity and positive predictive value, which fills a key gap to expedite opioid misuse identification in clinical settings.

In this study of patients with chronic pain, we demonstrated the ability of using regular expressions (an NLP technique) to automate the ABC instrument for identifying problematic opioid use within thousands of clinical notes. To our knowledge, this is the first attempt to automate the ABC instrument. We were able to achieve similar performance metrics on manually reviewed medical records between 2 separate health systems, which adds support for the generalizability of our approach. Future studies should compare our ABC–based NLP method with other NLP methods in the same dataset.^14,15^ Given the promising ability of our automated approach within a retrospective cohort, future work should evaluate the potential of prospective OUD identification in the clinical environment, including the opportunity to help train clinicians to identify patients at risk of OUD.

Clinical text and administrative billing codes each provide different information that can assist with the identification of problematic opioid use.^14,15,22^ However, the idea that a single domain of the EHR (eg, *ICD* codes) can adequately yield a valid phenotype is increasingly gaining scrutiny.^27,31,32^ Our study has demonstrated a scalable, reproducible way to extract meaningful information from clinical notes to augment other data domains within the EHR.

The ABC instrument has historically been completed by clinicians to assess risk of aberrant opioid use, with a threshold of 2 to 3 indicating potential inappropriate use.^16,33,34,35,36^ In this study, we found the threshold for the best performance (based on F1 scores) differed between the 2 sites, with a higher threshold in the validation site, which could be due to differences in documentation patterns between sites. For example, the validation site included about 3 times the number of notes as the primary site, which also increased the opportunity to observe regular expression matches. Differences in documentation patterns could be a result of higher illness severity in the validation site (ie, a dedicated pain clinic) compared with the primary site (ie, all patients with a chronic pain diagnosis). Supplement 1 contains a link to all software code necessary for replication. As other sites implement this automated version of the ABC, they should evaluate the optimal threshold for their clinicians’ unique documentation styles and cohort characteristics.

Items pertaining to increased narcotic use and medication agreements have been shown to be most aligned with clinical judgment (ie, “difficulty with using medication agreement,” “increased use of narcotics (since last visit),” “used more narcotics than prescribed,” and “patient indicated that s/he ‘needs’ or ‘must have’ analgesic meds^”^^16^). However, our most frequent matches pertained to expressions related to general addiction (ie, “Patient used illicit drugs or evidences problem drinking.”) and discussion of analgesic medications (ie, “Discussion of analgesic meds was the predominant issue of visit.”)^16^ The is perhaps unsurprising because SUD is highly comorbid with OUD and SUD behaviors are more frequently documented in the EHR than OUD behaviors.^37^ While we used a combined score, based on our finding of low item-level agreement of the individual ABC items there might be value in evaluating each item individually in future studies.

### Limitations

Our study also has its limitations. We used a single medical center to develop the instrument in a homogenous cohort of patients with chronic pain. While this instrument successfully generalized to a second site, keywords determined by OUD subject area experts might not represent the variety of language in a wide range of EHR notes. Additional input from external stakeholders and manual reviews of a larger corpus of notes could generate more expressions that would capture additional examples of representing ABC items in clinical notes.

While using EHR data during manual review might not be as robust as clinical interviews, we and others have identified useful OUD information via medical record reviews.^27,38^ While the 2 sites used different medical record review strategies, commonalities included key word search for OUD symptoms by individuals trained on medical terminology. The benefit of using key word search-based medical record review is that the methods are replicable vs relying only on expert review that typically results in a simple case/control designation, rather than explicit documentation of extracted details the reviewer used to make that determination.

Lastly, the ABC instrument was developed to identify OUD risk among patients with chronic pain, which might not generalize to people with OUD who obtain opioids without a prescription, including synthetic opioids. As psychometrically sound instruments are developed to identify OUD risk in patients without chronic pain, our automated approach can be expanded to include additional features.

## Conclusions

We leveraged the ABC, a publicly available, valid, and reliable instrument, for developing our text-based scoring system. Benefits of this method are interpretability (ie, one can review examples in the medical record that match a regular expression), generalizability to other organizations given it can be implemented in multiple software programs, and outperformance of diagnostic codes. Most immediately, this automated approach can serve as a superior alternative to diagnostic codes in research endeavors identifying OUD prevalence at a population level. Eventually, advances in this area will continue to facilitate earlier identification of people at risk for and experiencing problematic opioid use, which will create new opportunities for studying long-term sequelae of opioid pain management.

## Notes

Supplement 1. eAppendix 1. Full list of ICD codes eAppendix 2. List of opioid-related terms eAppendix 3. List of negation-related terms eAppendix 4. Regular expressions eAppendix 5. Individual performance of each ABC item (separate Excel File) eFigure.

Supplement 2. Data sharing statement
