# Supplementary material for: Automating the Addiction Behaviors Checklist for Problematic Opioid Use Identification
Source: JAMA Psychiatry. 2025 Apr 9;82(6):591–8. doi: 10.1001/jamapsychiatry.2025.0424 (PMC11983290; doi:10.1001/jamapsychiatry.2025.0424)
Supplement: Supplement 1. — eAppendix 1. Full list of ICD codes eAppendix 2. List of opioid-related terms eAppendix 3. List of negation-related terms eAppendix 4. Regular expressions eAppendix 5. Individual performance of each ABC item (separate Excel File) eFigure. [file jamapsychiatry-e250424-s001.pdf]

Supplemental Online Content

Chatham AH, Bradley ED, Troiani V, et al. Automating the addiction behaviors checklist for problematic opioid use identification. *JAMA Psych*. Published online April 9, 2025. doi:10.1001/jamapsychiatry.2025.0424

eAppendix 1. Full list of ICD codes

eAppendix 2. List of opioid-related terms

eAppendix 3 List of negation-related terms

eAppendix 4. Regular expressions

eAppendix 5. Individual performance of each ABC item

eFigure 1

This supplemental material has been provided by the authors to give readers additional information about their work.

© 2025 American Medical Association. All rights reserved, including those for text and data mining, AI training, and similar technologies.

Appendix A

Full list of ICD codes

Chronic Pain

ICD-9-CM: '338.2', '338.21', '338.22', '338.28', '338.29'

ICD-10-CM: 'G89.2', 'G89.21', 'G89.22', 'G89.28', 'G89.29'

Opioid Use Disorder

ICD-9-CM: '304', '304.0', '304.00', '304.01', '304.02', '304.03', '304.7', '304.70', '304.71', '304.72', '304.73', '305.5', '305.50', '305.51', '305.52', '305.53'

ICD-10-CM: 'F11','F11.1','F11.10', 'F11.120','F11.122','F11.129', 'F11.14', 'F11.150','F11.151','F11.159', 'F11.181','F11.182','F11.188', 'F11.19', 'F11.2','F11.20','F11.21','F11.220','F11.221','F11.222','F11.229', 'F11.23','F11.24','F11.250','F11.251','F11.259','F11.281','F11.282','F11.288','F11.29'

ICD-10: 'F11.0','F11.1','F11.2','F11.3','F11.4','F11.5','F11.7','F11.9'

Substance Use Disorder

ICD-9-CM: '292', '292.0', '292.1', '292.11', '292.12', '292.2', '292.8', '292.82', '292.83', '292.84', '292.85', '292.89', '292.9', '304', '304.0', '304.00', '304.01', '304.02', '304.03', '304.1', '304.10', '304.11', '304.12', '304.13', '304.2', '304.20', '304.21', '304.22', '304.23', '304.3', '304.30', '304.31', '304.32', '304.33', '304.4', '304.40', '304.41', '304.42', '304.43', '304.5', '304.50', '304.51', '304.52', '304.53', '304.6', '304.60', '304.61', '304.62', '304.63', '304.7', '304.70', '304.71', '304.72', '304.73', '304.8', '304.80', '304.81', '304.82', '304.83', '304.9', '304.90', '304.91', '304.92', '304.93', '305', '305.2', '305.20', '305.21', '305.22', '305.23', '305.3', '305.30', '305.31', '305.32', '305.33', '305.4', '305.40', '305.41', '305.42', '305.43', '305.5', '305.50', '305.51', '305.52', '305.53', '305.6', '305.60', '305.61', '305.62', '305.63', '305.7', '305.70', '305.71', '305.72', '305.73', '305.8', '305.80', '305.81', '305.82', '305.83', '305.9', '305.90', '305.91', '305.92', '305.93', '306', '357.6', '648.3', '648.30', '648.31', '648.32', '648.33', '648.34', '965.00', '965.01', '965.02'

ICD-10-CM:
'F11','F11.1','F11.10','F11.120','F11.122','F11.129','F11.14','F11.150','F11.151','F11.159','F11.181','F11.182','F11.188','F11.19','F11.2','F11.20','F11.21','F11.220','F11.221','F11.222','F11.229','F11.23','F11.24','F11.250','F11.251','F11.259','F11.281','F11.282','F11.288','F11.29','F11.90','F11.920','F11.922','F11.929','F11.93','F11.94','F11.950','F11.951','F11.959','F11.981','F11.982','F11.988','F11.99','F12','F12.1','F12.10','F12.120','F12.122','F12.129','F12.150','F12.151','F12.159','F12.180','F12.188','F12.19','F12.2','F12.20','F12.21','F12.220','F12.221','F12.222','F12.229','F12.250','F12.251','F12.259','F12.280','F12.288','F12.29','F12.90','F12.920','F12.922','F12.929','F12.950','F12.951','F12.959','F12.980','F12.988','F12.99','F13','F13.10','F13.120','F13.129','F13.14','F13.150','F13.151','F13.159','F13.180','F13.181','F13.182','F13.188','F13.19','F13.20','F13.21','F13.220','F13.221','F13.229','F13.230','F13.231','F13.232','F13.239','F13.24','F13.250','F13.251','F13.259','F13.26','F13.27','F13.280','F13.281','F13.282','F13.288','F13.29','F13.90','F13.920','F13.929','F13.930','F13.931','F13.932','F13.939','F13.94','F13.950','F13.951','F13.959','F13.96','F13.97','F13.980','F13.981','F13.982','F13.988','F13.99','F14','F14.1','F14.10','F14.120','F14.122','F14.129','F14.14','F14.150','F14.151','F14.159','F14.180','F14.181','F14.182','F14.188','F14.19','F14.2','F14.20','F14.21','F14.220','F14.221','F14.222','F14.229','F14.23','F14.24','F14.250','F14.251','F14.259','F14.280','F14.281','F14.282','F14.288','F14.29','F14.90','F14.920','F14.922','F14.929','F14.94','F14.950','F14.951','F14.959','F14.980','F14.981','F14.982','F14.988','F14.99','F15','F15.10','F15.120','F15.122','F15.129','F15.14','F15.150','F15.151','F15.159','F15.180','F15.181','F15.182','F15.188','F15.19','F15.20','F15.21','F15.220','F15.221','F15.222','F15.229','F15.23','F15.24','F15.250','F15.251','F15.259','F15.280','F15.281','F15.282','F15.288','F15.29','F15.90','F15.920','F15.922','F15.929','F15.93','F15.94','F15.950','F15.951','F15.959','F15.980','F15.981','F15.982','F15.988','F15.99','F16','F16.1','F16.10','F16.120','F16.122','F16.129','F16.14','F16.150','F16.151','F16.159','F16.180','F16.183','F16.188','F16.19','F16.2','F16.20','F16.21','F16.220','F16.221','F16.229','F16.24','F16.250','F16.251','F16.259','F16.280','F16.283','F16.288','F16.29','F16.90','F16.920','F16.929','F16.94','F16.950','F16.951','F16.959','F16.980','F16.983','F16.988','F16.99','F17.203','F17.208','F17.209','F17.213','F17.218','F17.219','F17.223','F17.228','F17.229','F17.293','F17.298','F17.299','F18','F18.1','F18.10','F18.120','F18.129','F18.14','F18.150','

© 2025 American Medical Association. All rights reserved, including those for text and data mining, AI training, and similar technologies.

F18.151','F18.159','F18.17','F18.180','F18.188','F18.19','F18.2','F18.20','F18.21','F18.220','F18.221','F18.229','F18.24','F18.250','F18.251','F18.259','F18.27','F18.280','F18.288','F18.29','F18.90','F18.920','F18.929','F18.94','F18.950','F18.951','F18.959','F18.97','F18.980','F18.988','F18.99','F19.10','F19.120','F19.122','F19.129','F19.14','F19.150','F19.151','F19.159','F19.16','F19.17','F19.180','F19.181','F19.182','F19.188','F19.19','F19.20','F19.21','F19.220','F19.221','F19.222','F19.229','F19.230','F19.231','F19.232','F19.239','F19.24','F19.250','F19.251','F19.259','F19.26','F19.27','F19.280','F19.281','F19.282','F19.288','F19.29','F19.90','F19.920','F19.922','F19.929','F19.930','F19.931','F19.932','F19.939','F19.94','F19.950','F19.951','F19.959','F19.96','F19.97','F19.980','F19.981','F19.982','F19.988','F19.99','F55','F55.0','F55.1','F55.2','F55.3','F55.4','F55.8','O99.32','O99.320','O99.321','O99.322','O99.323','O99.324','O99.325'

ICD-10:
'F10','F11.0','F11.1','F11.2','F11.3','F11.4','F11.5','F11.7','F11.9','F12.0','F12.1','F12.2','F12.5','F12.8','F12.9','F13.0','F13.1','F13.2','F13.3','F13.4','F14.0','F14.1','F14.2','F14.5','F14.9','F15.0','F15.1','F15.2','F15.3','F15.5','F15.8','F15.9','F16.1','F16.2','F16.3','F16.5','F16.8','F16.9','F55','T40.1','T40.3'

© 2025 American Medical Association. All rights reserved, including those for text and data mining, AI training, and similar technologies.

Appendix B

List of opioid-related terms

'pain med', 'opioid', 'opiod', '\bnarc', 'analges', 'suboxone', 'Avinza', 'codeine', 'dilaudid', 'fentanyl', 'hydrocodone', 'morphine', 'opana', 'opiate', 'oxycodone', 'oxycontin', 'oxymorphone', 'percocet', 'roxicodone', 'sufentanyl', 'vicodin', 'lortab', 'hydromorphone', 'abstral', 'actiq', 'alfentanil', 'arymo', 'ascomp', 'astramorph', 'avinza', 'belbuca', 'brompheniramine', 'bunavail', 'buprenex', 'buprenorphine', 'butalbital', 'butorphanol', 'butrans', 'capcof', 'carisoprodol', 'cheratussin', 'coditussin', 'conzip', 'demerol', 'dexbrompheniramine', 'dihydrocodeine', 'diskets', 'dolophine', 'durmorph', 'embeda', 'endacof', 'endocet', 'exalgo', 'fentora', 'fioricet', 'flowtuss', 'guaifenesin', 'histex', 'hycet', 'hycofenix', 'hydrocodone', 'hydromorphone', 'hysingla', 'ibudone', 'infumorph', 'iophen', 'iorinal', 'kadian', 'lazanda', 'levorphanol', 'lorcet', 'lotruss', 'meperidine', 'methadone', 'methadose', 'morphabond', 'morphine', 'ms contin', 'nalbuphine', 'nalocet', 'ninjacof', 'nucynta', 'obredon', 'opana', 'opium', 'orco', 'oxaydo', 'oxecta', 'oxycodone', 'panlor', 'paregoric', 'pentazocine', 'percocet', 'phenylhistine', 'primlev', 'pro-clear', 'probuphine', 'promethazine', 'psuedoephedrine', 'relcof', 'remifentanil', 'reprexain', 'rezira', 'robafen', 'roxicodone', 'rydex', 'suboxone', 'subsys', 'sufentanil', 'synalgos', 'talwin', 'tapentado', 'tramadol', 'trezix', 'triplidine', 'trymine', 'tusnel', 'tussicpas', 'ultiva', 'ultracet', 'ultram', 'verdrocet', 'vicodin', 'vicoprofen', 'virtussin', 'xartemix', 'xodol', 'xtampza', 'zamicet', 'zodryl', 'zubsolv', 'zutripro', 'zylon'

© 2025 American Medical Association. All rights reserved, including those for text and data mining, AI training, and similar technologies.

Appendix C

List of negation-related terms

'\bno\b', '\bnot\b', 'denies', 'denial', 'doubt', 'never', 'negative for'

© 2025 American Medical Association. All rights reserved, including those for text and data mining, AI training, and similar technologies.

Appendix D

Python-based software code for implementing the regular expressions used to generate the Addiction Behaviors Checklist (ABC) instrument. The following code can be directly copied and pasted into a python script to serve as a dictionary object to reference each ABC item's label/name/description, the regular expression pattern, and whether to perform additional checks such as nearby opioid mentions, negation, and common false positive terms.

```
checklist = {'1a' : {'lab' : 'Since last visit: #1 "Patient used illicit drugs or evidences problem drinking" #1a Illicit drugs',
                    'pat' : r'((illicit drug)|mariju|cocai|heroin|polysubst|methamphetamine|amphetamine|\becstasy|(street drugs)|IVDU|(IV drug))',
                    'col_name' : 'illicit_drugs',
                    'opioid' : True,
                    'negation' : True,
                    'preview' : False},
            '1b' : {'lab' : '#1b Problematic alcohol use',
                    'pat' : r'((problem)([^\.]{1,10})(drink|alcoh|etoh))|(((drink|alcoh|etoh))([^\.]{1,10})(abuse|addic|ism|dependence)(?![^\.]{1,10}(father|mother)))',
                    'col_name' : 'problem_drinking',
                    'opioid' : False,
                    'negation' : True,
                    'preview' : False,
                    'common_fp' : ['mother', 'sister', 'father', 'brother', 'aunt', 'uncle']},
            '1c' : {'lab' : '1c. DUIs',
                    'pat' : r'(\bDUI\b)',
                    'col_name' : 'dui',
                    'opioid' : False,
                    'negation' : True,
                    'preview' : False},
            '2' : {'lab' : '#2 "Patient has hoarded meds"',
                    'pat' : r'(\bhoard|stash|(left over)|\bstoring|stockpil)',
                    'col_name' : 'hoarding',
                    'opioid' : True,
                    'negation' : True,
                    'preview' : False,
                    'common_fp' : ['hoarder']},
             '3' : {'lab' : '#3 "Patient used more narcotic than prescribed"',
                    'pat' : r'((more+(?=.{1,50}than presc))|(ta?o?o?ke?i?n?g? extra(?![^\.]{1,10}strength)))',
                    'col_name' : 'more_narcotic',
                    'opioid' : True,
                    'negation' : True,
                    'preview' : False},
             '4' : {'lab' : '#4 "Patient ran out of meds early"',
                    'pat' :  r'(((running|ran) \bout\b(?=.{1,50}earl))|((\bout\b(?=.{1,50}earl)))|(too early to refill)|(refill early))',
                    'col_name' : 'ran_out_early',
                    'opioid' : True,
                    'negation' : True,
                    'preview' : False,
                    'common_fp' : ['out of bed']},
             '5a' : {'lab' : '#5a "Patient has increased use of narcotics" 5a. Increased narcotic/opioid use',
                    'pat' : r'((increased|increasing) use)',
                    'col_name' : 'increased_op',
                    'opioid' : True,
```

© 2025 American Medical Association. All rights reserved, including those for text and data mining, AI training, and similar technologies.

```
            'negation' : True,
            'preview' : False},
    '5b' : {'lab' : '#5b Dose escalation (alternative to increased use)',
            'pat' : r'((escalate?d?i?o?n?(?=((.{1,10})dos)))|(dose?a?g?e?(?=((.{1,10})escalat))))',
            'col_name' : 'escalation_op',
            'opioid' : True,
            'negation' : True,
            'preview' : False},
    '6' : {'lab' : '#6 "Patient used analgesics PRN when prescription is for time contingent use"',
            'pat' : r'(not?n? ?-?adher)|(non?t?-? ?complian)',
            'col_name' : 'prn',
            'opioid' : True,
            'negation' : False,
            'preview' : False,
            'common_fp' : ['mepitel nonadherent dressing', 'diet', 'regimen']},
    '7a' : {'lab' : '#7 "Patient received narcotics from more than one provider"',
            'pat' : r'(more than one|multiple)(?=((.{1,50}(provider|doctor|pharmac|prescrib))))',
            'col_name' : 'multiple_sources_x',
            'opioid' : True,
            'negation' : True,
            'preview' : False,
            'common_fp' : ['diagnoses']},
    '7b' : {'lab' : 'Alternate "More than one provider" Search',
            'pat' : r'(more than one|multiple)(?=((.{1,50}(provider|doctor|pharmac|prescrib))))',
            'col_name' : 'multiple_sources_y',
            'opioid' : True,
            'negation' : True,
            'preview' : False,
            'common_fp' : ['diagnoses']},
    '8' : {'lab' : '#8 "Patient bought meds on the streets"',  #alternate: r'((med|drug).{1,10}(on the street))|((on the
street).{1,10}(med|drug))'
            'pat' : r'(on the street)',
            'col_name' : 'bought_on_street',
            'opioid' : True,
            'negation' : True,
            'preview' : False},
    '9' : {'lab' : '#9 "Patient appears sedated or confused (e.g., slurred speech, unresponsive)"',
            'pat' : r'(sedated|confused|(slurred speech)|unresponsive)',
            'col_name' : 'sedated',
            'opioid' : True,
            'negation' : True,
            'preview' : False},
    '10' : {'lab' : '#10 "Patient expresses worries about addiction"',
            'pat' : r'(worr.{1,25}addic)',
            'col_name' : 'worries_about_addiction',
            'opioid' : False,
            'negation' : True,
            'preview' : False},
    '11a' : {'lab' : '#11 "Patient expressed a strong preference for a specific type of analgesic or a specific route
of administration"',
            'pat' : r'((patient|pt) (request|prefer|(preferr?s? to take)))',
            'col_name' : 'strong_preference',
            'opioid' : True,
            'negation' : True,
            'preview' : False,
            'common_fp' : ['pharm']},
```

© 2025 American Medical Association. All rights reserved, including those for text and data mining, AI training, and similar technologies.

```
'11b' : {'lab' : '#11b Strong Preference, IV',
        'pat' : 'r((patient|pt) (request|prefer|(prefers to take))([^\.]{1,10}IV))',
        'col_name' : 'strong_preference_IV',
        'opioid' : False,
        'negation' : False,
        'preview' : False},
'12a' : {'lab' : '#12a "Patient expresses concern about future availability of narcotic"', #change take to tak and
add opioid detection
        'pat' : r'(((take)[^\.]{1,25})(away))',
        'col_name' : 'future_availability_1',
        'opioid' : True,
        'negation' : True,
        'preview' : False,
        'common_fp' : ['disp', 'rfl', 'allerg']},
'12b' : {'lab' : '#12b Future Availability: Loss of medication',
        'pat' : r'((((won|can)\'?t)[^\.]{1,25}(get))|(loss of medication))',
        'col_name' : 'future_availability_2',
        'opioid' : True,
        'negation' : True,
        'preview' : False},
'13' : {'lab' : '#13 "Patient reports worsened relationships with family"',
        'pat' : r'(family (dynamics|problem))|(relationship
problem)|((lost|(took))[^\.]{1,25}(kids|children|\bson|daughter))',
        'col_name' : 'worsened_relationships',
        'opioid' : True,
        'negation' : True,
        'preview' : False},
'14' : {'lab' : '#14 "Patient misrepresented analgesic prescription or use"',
        'pat' : r'(misrepresent|(lied? about)|(\blying about))',
        'col_name' : 'misrep_use',
        'opioid' : True,
        'negation' : False,
        'preview' : False},
'15' : {'lab' : '#15 "Patient indicated she or he ‘‘needs’’ or ‘‘must have’’ analgesic meds"',
        'pat' : '((must have)|demands)',
        'col_name' : 'needs_must_have' ,
        'opioid' : True,
        'negation' : False,
        'preview' : False,
        'common_fp' : [r'[0-9]']},
'16' : {'lab' : '#16 "Discussion of analgesic meds was the predominant issue of visit"',
        'pat' : r'(pain med)|opioid|opiod|
narc|analges|suboxone|Avinza|codeine|dilaudid|fentanyl|hydrocodone|morphine|opana|opiate|oxycodone|oxycontin|o
xymorphone|percocet|roxicodone|sufentanyl|vicodin|lortab|hydromorphone|abstral|actiq|alfentanil|arymo|ascomp|astr
amorph|avinza|belbuca|brompheniramine|bunavail|buprenex|buprenorphine|butalbital|butorphanol|butrans|capcof|cari
soprodol|cheratussin|coditussin|conzip|demerol|dexbrompheniramine|dihydrocodeine|diskets|dolophine|durmorph|em
beda|endacof|endocet|exalgo|fentora|fioricet|flowtuss|guaifenesin|histex|hycet|hycofenix|hydrocodone|hydromorphon
e|hysingla|ibudone|infumorph|iophen|iorinal|kadian|lazanda|levorphanol|lorcet|lotruss|meperidine|methadone|methad
ose|morphabond|morphine|ms
contin|nalbuphine|nalocet|ninjacof|nucynta|obredon|opana|opium|orco|oxaydo|oxecta|oxycodone|panlor|paregoric|pen
tazocine|percocet|phenylhistine|primlev|pro-
clear|probuphine|promethazine|psuedoephedrine|relcof|remifentanil|reprexain|rezira|robafen|roxicodone|rydex|subox
one|subsys|sufentanil|synalgos|talwin|tapentado|tramadol|trezix|triplidine|trymine|tusnel|tussicpas|ultiva|ultracet|ultra
m|verdrocet|vicodin|vicoprofen|virtussin|xartemix|xodol|xtampza|zamicet|zodryl|zubsolv|zutripro|zylon',
        'col_name' : 'opioid_tag',
        'opioid' : True,
```

© 2025 American Medical Association. All rights reserved, including those for text and data mining, AI training, and similar technologies.

```
                'negation' : True,
                'preview' : False,
                'common_fp' : ['disp', 'rfl', 'allerg', 'mg']},
        '17' : {'lab' : '#17 "Patient exhibited lack of interest in rehab or self-management"',
                'pat' : r'((not|no[^\.(need)]{1,25}(interest|seen|want|go))|resistant|dislike|refuse)([^\.]{1,25})(rehab|(pain management)|(pain clinic))',
                'col_name' : 'lack_interest_rehab',
                'opioid' : True,
                'negation' : False,
                'preview' : False},
        '18a' : {'lab' : '#18a "Patient reports minimal/inadequate relief from narcotic analgesic"',
                'pat' : r'(not?\b|minimal|limited)([^\.]{1,25})(relief (from|with|w/t|))',
                'col_name' : 'minimal_relief_x',
                'opioid' : True,
                'negation' : False, # changed to False when Eli originally had True but that would've created a double-negative
                'preview' : False},
        '18b' : {'lab' : 'Patient reports minimal/inadequate relief: Tolerance',
                'pat' : r'(\btoleran)',
                'col_name' : 'tolerance',
                'opioid' : True,
                'negation' : True,
                'preview' : False,
                'common_fp' : ['activity']},
        '19' : {'lab' : '#19 "Patient indicated difficulty with using medication agreement"',
                'pat' : r'((medication|med|opioi?d|narcotic) (agreement|contract) (breach|violat|problem|issue|fail))|((violat|problem|issue|fail|breach)[^\.]{1,25}(medication|med|opioi?d|narcotic) (agreement|contract))',
                'col_name' : 'med_agreement',
                'opioid' : False,
                'negation' : False,
                'preview' : False},
        '20' : {'lab' : '#20 (Other) "Significant others express concern over patient’s use of analgesics"',
                'pat' : r'(wife|\bmother|father|husband|daughter|aunt|uncle|\bson|(significant other)|(family member))([^\.]{1,25})(concern|worr)',
                'col_name' : 'SO_concern',
                'opioid' : True,
                'negation' : True,
                'preview' : False},
        }
```

© 2025 American Medical Association. All rights reserved, including those for text and data mining, AI training, and similar technologies.

Appendix E

Individual performance of each Addiction Behaviors Checklist (ABC) item.

See separate Excel file for item-level performance of the automated ABC checklist where the data can be sorted by any column.

Primary Site Performance: When evaluating each regular expression-based ABC item against the Test Set, sensitivity ranged 0.00-0.90, specificity ranged 0.43-1.00, positive predictive value ranged 0.00-1.00, negative predictive value ranged 0.49-0.60, and F1-scores ranged 0.00-0.73. The ABC item with the highest F1-score performance was “Patient used illicit drugs or evidences problem drinking.”

Validation Site Performance: When evaluating each regular expression-based ABC item, sensitivity ranged 0.00-1.00, specificity ranged 0.00-1.00, positive predictive value ranged 0.00-1.00, negative predictive value ranged 0.00-0.93, and F1 scores ranged 0.00-0.49. The ABC item with the highest F1-score performance was “Patient reports minimal/inadequate relief from narcotic analgesic.”

Definitions: We calculated the sensitivity (i.e., recall - proportion of cases with an NLP match), specificity (proportion of controls without an NLP match), positive predictive value [PPV] (i.e., precision - proportion of NLP matches that were cases), negative predictive value ([NPV] proportion of NLP non-matches that were controls), and F1-score (a single measure of predictive performance combining the sensitivity and positive predictive values) of our regular expression scoring system against the manually adjudicated labels in the Test Set (n=100 at each site). While AUCs are frequently used in the biomedical and clinical literature to represent performance of a diagnostic test/model, reporting an AUC depends on calculating specificity, which can be misleading in information retrieval studies, such as those using NLP methods. By focusing on sensitivity (recall) and positive predictive value (precision), the measures emphasize identification of positive cases, which can be particularly helpful when datasets do not have a case-control balance.

© 2025 American Medical Association. All rights reserved, including those for text and data mining, AI training, and similar technologies.

Item Number	Item Description	Vanderbilt University Medical Center: No. (%) of Patients with a Match, Entire Cohort (n = 8063)	Vanderbilt: No. (%) of Patients with a Match, Test Set (n = 99)	Vanderbilt: Performance in Test Set, *Sensitivity*	*Specificity*	*PPV*	*NPV*	*F1*	Geisinger: No. (%) of Patients with a Match, Test Set (n = 100)	Geisinger: Performance in Test Set, *Sensitivity*	*Specificity*	*PPV*	*NPV*	*F1*
1	Patient used illicit drugs or evidences problem drinking	1454 (18.0)	21 (21.2)	0.38	0.96	0.9	0.6	0.54	72 (72.0)	0.91	0.33	0.28	0.93	0.43
2	Patient has hoarded meds	130 (1.6)	1 (1.0)	0.02	1	1	0.5	0.04	9 (9.0)	0.18	0.94	0.44	0.8	0.26
3	Patient used more narcotic than prescribed	163 (2.0)	1 (1.0)	0.02	1	1	0.5	0.04	5 (5.0)	0.14	0.97	0.6	0.8	0.22
4	Patient ran out of meds early	168 (2.1)	2 (2.0)	0.04	1	1	0.51	0.08	6 (6.0)	0.27	1	1	0.83	0.43
5	Patient has increased use of narcotic	124 (1.5)	1 (1.0)	0.02	1	1	0.5	0.04	3 (3.0)	0.09	0.99	0.67	0.79	0.16
6	Patient used analgesics PRN when prescription is for time contingent use	251 (3.1)	5 (5.1)	0.1	1	1	0.52	0.18	3 (3.0)	0.09	0.99	0.67	0.79	0.16
7	Patient received narcotics from more than one provider	231 (2.9)	4 (4.0)	0.08	1	1	0.52	0.15	5 (5.0)	0.14	0.97	0.6	0.8	0.22
8	Patient bought meds on the streets	31 (0.4)	0 (0.0)	0	1	0	0.49	N/A	1 (1.0)	0.05	1	1	0.79	0.09
9	* Patient appears sedated or confuses (e.g., slurred speech, unresponsive)	1003 (12.4)	17 (17.2)	0.26	0.92	0.76	0.55	0.39	16 (16.0)	0.36	0.9	0.5	0.83	0.42
10	Patient expresses worries about addiction	18 (0.2)	1 (1.0)	0.02	1	1	0.5	0.04	5 (5.0)	0.05	0.95	0.2	0.78	0.07
11	Patient expressed a strong preference for a specific type of analgesic or a specific route of administration	1022 (12.7)	10 (10.1)	0.2	1	1	0.55	0.33	53 (53.0)	0.77	0.54	0.32	0.89	0.45
12	Patient expresses concern about future availability of narcotic	241 (3.0)	3 (3.0)	0.06	1	1	0.51	0.11	10 (10.0)	0.23	0.94	0.5	0.81	0.31
13	Patient reports worsened relationships with family	16 (0.2)	0 (0.0)	0	1	0	0.49	N/A	2 (2.0)	0.09	1	1	0.8	0.17
14	Patient misrepresented analgesic prescription or use	2 (0.0)	0 (0.0)	0	1	0	0.49	N/A	0 (0.0)	0	1	N/A	0.78	0
15	† Patient indicated she or he ‘‘needs’’ or ‘‘must have’’ analgesic meds	66 (0.8)	0 (0.0)	0	1	0	0.49	N/A	4 (4.0)	0.05	0.96	0.25	0.78	0.08
16	Discussion of analgesic meds was the predominant issue of visit	6171 (76.5)	73 (73.7)	0.9	0.43	0.62	0.81	0.73	100 (100.0)	1	0	0.22	N/A	0.36
17	Patient exhibited lack of interest in rehab or self-management	117 (1.5)	3 (3.0)	0.06	1	1	0.51	0.11	10 (10.0)	0.23	0.94	0.5	0.81	0.31
18	Patient reports minimal/inadequate relief from narcotic analgesic	1025 (12.7)	20 (20.2)	0.34	0.94	0.85	0.58	0.49	48 (48.0)	0.77	0.6	0.35	0.9	0.49
19	Patient indicated difficulty with using medication agreement	62 (0.8)	0 (0.0)	0	1	0	0.49	N/A	3 (3.0)	0.09	0.99	0.67	0.79	0.16
20	Significant others express concern over patient’s use of analgesics	137 (1.7)	1 (1.0)	0.02	1	1	0.5	0.04	2 (2.0)	0.05	0.99	0.5	0.79	0.08

* Future work could focus on excluding mentions near other sedating interventions (e.g., patient-controlled analgesia in the hospital setting). † Regular expressions were unable to capture the intent of this item.

© 2025 American Medical Association. All rights reserved, including those for text and data mining, AI training, and similar technologies.

eFigure 1. Recall-precision curve of combined score from regular expression-based ABC instrument compared to manual review (solid lines) and ICD codes (dashed lines).

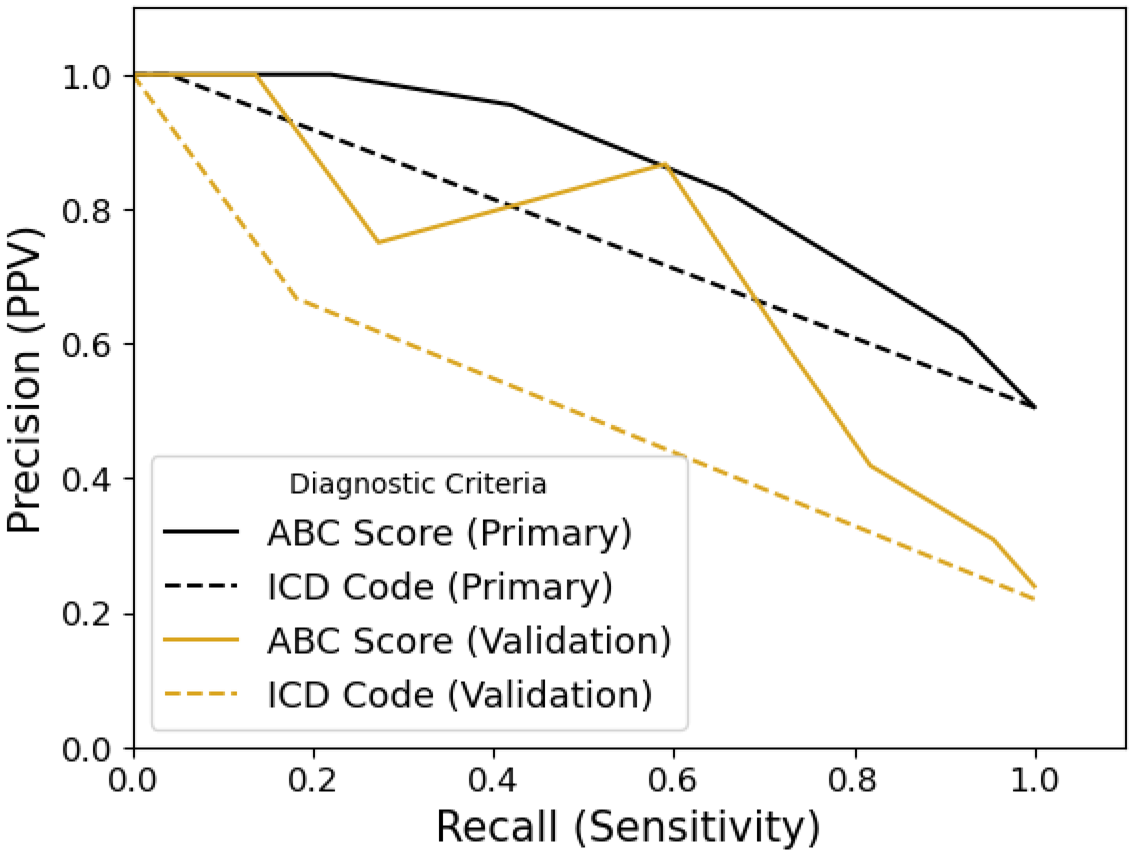

Note: The frequently changing direction of the ABC Score in the Validation Site is a result of the changing performance at different ABC Score thresholds. The sensitivity (recall) and precision (positive predictive value) of the total ABC score were higher than that of the ICD codes, as compared to manual review.

© 2025 American Medical Association. All rights reserved, including those for text and data mining, AI training, and similar technologies.
