# Supplementary material for: Automating the Addiction Behaviors Checklist for Problematic Opioid Use Identification
Source: JAMA Psychiatry. 2025 Apr 9;82(6):591–8. doi: 10.1001/jamapsychiatry.2025.0424 (PMC11983290; doi:10.1001/jamapsychiatry.2025.0424)
Supplement: Supplement 2. — Data sharing statement [file jamapsychiatry-e250424-s002.pdf]

Data Sharing Statement

Chatham. Automating the Addiction Behaviors Checklist for Problematic Opioid Use Identification. *JAMA Psychiatry*. Published April 09, 2025.
doi:10.1001/jamapsychiatry.2025.0424

Data
Data available: No
